# Gender differences in one-year unplanned readmissions in atrial fibrillation: Trends from a conflict-stricken country

**DOI:** 10.1177/02676591251407291

**Published:** 2025-12-03

**Authors:** Ibrahim Antoun, Alkassem Alkhayer, Alamer Alkhayer, Aref Jalal Eldin, Georgia R Layton, Riyaz Somani, G André Ng, Mustafa Zakkar

**Affiliations:** 1 Department of Cardiovascular Sciences, 150459 University of Leicester, Leicester, UK; 2 Faculty of Medicine, University of Aleppo, Aleppo, Syria; 3 Department of Cardiology, Glenfield Hospital, University Hospitals of Leicester NHS Trust, Leicester, UK; 4 Faculty of Medicine, 594809 University of Latakia’s Hospital, Latakia, Syria; 5 Department of Cardiac Surgery, Glenfield Hospital, University Hospitals of Leicester NHS Trust, Leicester, UK; 6 Leicester British Heart Foundation Centre of Research Excellence, Leicester, UK; 7 Leicester National Institute for Health and Care Research Biomedical Research Centre, Leicester, UK; 8 Faculty of Medicine, University of Damascus, Damascus, Syria

**Keywords:** atrial fibrillation, readmission, Syria, conflict, outcomes, gender

## Abstract

**Introduction:**

Atrial fibrillation (AF) is the most common arrhythmia worldwide, yet long-term outcomes in conflict-affected regions are poorly understood. Gender-based disparities in AF outcomes have been reported in high-income countries but remain unexplored in fragile healthcare systems.

**Methods:**

We conducted a retrospective cohort study at Latakia's University Hospital in Latakia, Syria between June/2021-November/2023. Adult patients admitted with primary AF were followed for 1 year to assess unplanned readmissions. Data on unplanned readmissions were defined as non-elective hospitalisations occurring within 1 year after index discharge. These were unscheduled admissions, usually prompted by recurrence of symptoms, acute decompensation, or cardiovascular events. Data were collected from medical records.

**Results:**

Of the included 657 patients (52% male, median age 60 years), 422 (64%) had at least one unplanned readmission within 1 year. Cardiac causes accounted for 67% of readmissions, with recurrent AF responsible for 75% of those. Females had higher all-cause (73% vs 56%) and cardiovascular (53% vs 34%) readmission rates than males (both *p* < 0.001). On multivariable analysis, independent predictors of readmission included female sex (HR 1.7, 95% CI 1.4–2.0), age ≥60 (HR 3.7, 95% CI 2.9–4.6), diabetes mellites (DM) (HR 1.5, 95% CI 1.2–1.8), and congestive heart failure (CCF) (HR 3.3, 95% CI 2.6–4.6). Females were more likely to have two (44%) or three or more (44%) readmissions than males.

**Conclusions:**

One-year readmissions after AF admission were high, particularly among females. Female gender was an independent risk factor, highlighting the need for gender-sensitive follow-up strategies in resource-limited settings.

## Introduction

Atrial fibrillation (AF) is the most common type of arrhythmia worldwide, and its prevalence in middle-to low-income countries is likely underestimated.^1^ Although AF in the developed world is well studied, there is little data on AF management patterns in the Middle East.^2^

One of these countries, Syria, has been suffering from a conflict for the past 14 years. This has left the country deprived of healthcare funding and resources, which was exacerbated during the COVID-19 pandemic and cholera outbreak.^3,4^ Less than half the hospitals operate at full capacity, and more than half of the healthcare workers are forced to flee the country due to escalating conflict.^5^ AF management in Syrian hospitals during the current economic and political turmoil is unclear, with a paucity of published inpatient figures and outcomes originating from Syrian healthcare. A real-world depiction of the current AF care in the context of resource limitations can help manage and allocate resources by recognising remediable deficiencies and identifying practical and reasonable solutions that can be enforced.^6^ Although advances in AF management have improved symptom control, readmission rates continue to increase and have been a primary source of financial constraints on healthcare economies related to AF.^7^ Particularly for Syria, following up on patients after initial admissions associated with AF is challenging due to limited healthcare resources and damaged infrastructure.^5^ Readmission rates and predictors were studied in developed countries^7–13^ but not in developing and conflict-stricken countries.

Moreover, gender differences in AF outcomes have gained attention in recent years. Evidence from high-income countries suggests that females with AF often experience worse symptoms, higher rates of adverse outcomes and are less likely to receive rhythm control strategies.^14,15^ These inequities may be amplified in low-resource contexts, where cultural norms, healthcare access, and socioeconomic constraints disproportionately affect women. However, no prior study has examined sex-specific differences in long-term AF outcomes within a conflict-affected country.

This study aimed to evaluate the rate, aetiologies, and predictors of 1-year unplanned readmissions after index admission for primary AF in Syria, with a specific focus on gender differences in outcomes.

## Methods

### Design and data collection

This is a retrospective single-centre observational cohort study conducted at Latakia's University Hospital in Latakia, Syria, from June 2021 to November 2023. The hospital is a large, government-operated public institution affiliated with Tishreen University, serving as the primary healthcare centre for the city and its surrounding areas. The hospital has 860 beds and provides free healthcare to Syrian citizens. The hospital cares for approximately 50,000–60,000 inpatients annually, with more outpatients seeking care in various departments. Although located in Latakia, a coastal region less directly impacted by frontline fighting, Tishreen University Hospital functions as a major public tertiary referral centre providing free care to all Syrian citizens. The hospital serves both the local population and patients displaced from other conflict-affected areas. The study included patients aged 18 years or older who were treated for AF as the primary diagnosis during their index admission. Patients with secondary AF and missing data for sex and age were excluded. Patients were followed for 1 year after discharge from their initial admission to track readmissions. The study’s data sources included hospital records, both paper and electronic. The medical or cardiology consultant determined the causes of the index admission and readmissions. The research reported in this article adhered to the Declaration of Helsinki. The study was conducted as part of an audit approved by the hospital board and involved prospective analysis of retrospectively collected anonymised data. Therefore, the ethical committee of Tishreen University Hospital waived the need for consent. The study was reported in accordance with the STROBE statement.^16^

### Outcomes

The primary outcome of our study was 1-year readmissions and their etiologies in males and females. Readmissions were defined as unplanned, non-elective hospitalisations not scheduled in advance. A secondary analysis explored predictors of 1-year readmissions and the frequency of readmissions in males and females.

### Statistical analysis

Continuous variables are expressed as medians and interquartile ranges (IQR), while categorical variables are expressed as counts and percentages (%). Pearson’s χ 2 or Fisher’s exact test was used to compare categorical variables between groups, while the Student's *t*-test was used to compare continuous variables.

Kaplan-Meier models and Cox regression were used to investigate the relationship between variables (including gender) and the probability of readmission within 1 year. We hypothesised that specific demographics and comorbidities would affect the 1-year readmission probability. Therefore, a base model was constructed to assess the incremental value of comorbidities significantly associated with 1-year readmission. Statistically significant comorbidities identified in the univariate analysis were included in the multivariable analysis. A 2-sided *p*-value <0.05 was considered statistically significant. Statistical analysis was performed using GraphPad Prism V10.3 for Mac (San Diego, California, USA).

## Results

### Baseline characteristics

Over the study period, an estimated 150,000 patients visited the emergency department, of whom 340 (22%) were male, with a median age of 60. A total of 657 had an index admission with primary AF. Among these, 422 (64%) experienced an unplanned readmission within 1 year after discharge from the index hospitalisation. The demographics for males and females are shown in Table 1. Compared to males, females were older (65 vs 57 years, *p* < 0.001), had a higher proportion of patients with ischemic heart disease (IHD) (41% vs 12%; *p* < 0.001) and diabetes mellitus (DM) (26% vs 18%; *p* < 0.001). The remaining demographics and treatment approaches were similar between males and females.

**Table 1.** Baseline characteristics of the study cohort in males and females.

	Overall (657)	Male (*n* = 340)	Female (*n* = 317)	*p*-value
Age (years)	60 (54–67)	57 (51–57)	65 (55–70)	<0.001
Hypertension	221 (34%)	115 (34%)	106 (33%)	0.88
Ischaemic heart disease	171 (26%)	41 (12%)	130 (41%)	<0.001
Diabetes mellitus	145 (22%)	88 (26%)	57 (18%)	<0.001
Cerebrovascular disease	123 (19%)	27 (21%)	15 (16%)	0.23
Congestive heart failure	123 (19%)	69 (20%)	54 (17%)	0.06
Thyroid disease	22 (3%)	15 (4%)	7 (2%)	0.13
CHA2DVA score	2 (1–3)	1 (1–2)	2 (2–3.5)	<0.001
Active malignancy	44 (7%)	19 (6%)	25 (8%)	0.43
Chronic liver failure	50 (8%)	20 (6%)	30 (9%)	0.22
Chronic lung disease	72 (11%)	33 (10%)	39 (12%)	0.03
Chronic kidney failure	58 (8%)	29 (9%)	29 (9%)	0.61
Rate control	657 (100%)	340 (100%)	317 (100%)	0.99
Rhythm control^a^	150 (23%)	82 (24%)	68 (21%)	0.45

IQR: interquartile ranges.

^a^Amiodarone was the only rhythm control method used.

### Aetiologies of 1-year readmissions

Figure 1 illustrates the aetiologies of 1-year readmission and compares them between males and females. Cardiac conditions were the most common cause of 1-year readmission (67%). Of cardiac conditions, the most common condition was AF (75%), followed by decompensated CCF (10%) and myocardial infarction (7%). The most common noncardiac causes of readmission were trauma (6%), infection, pulmonary causes, and cerebrovascular events, at 5% each. Compared to males, females had more all-cause readmissions and cardiovascular readmissions (230 [55%] vs 192 [45%], *p* < 0.001 and 167 [59%] vs 116 [41%], *p* < 0.001, respectively).

**Figure 1.**
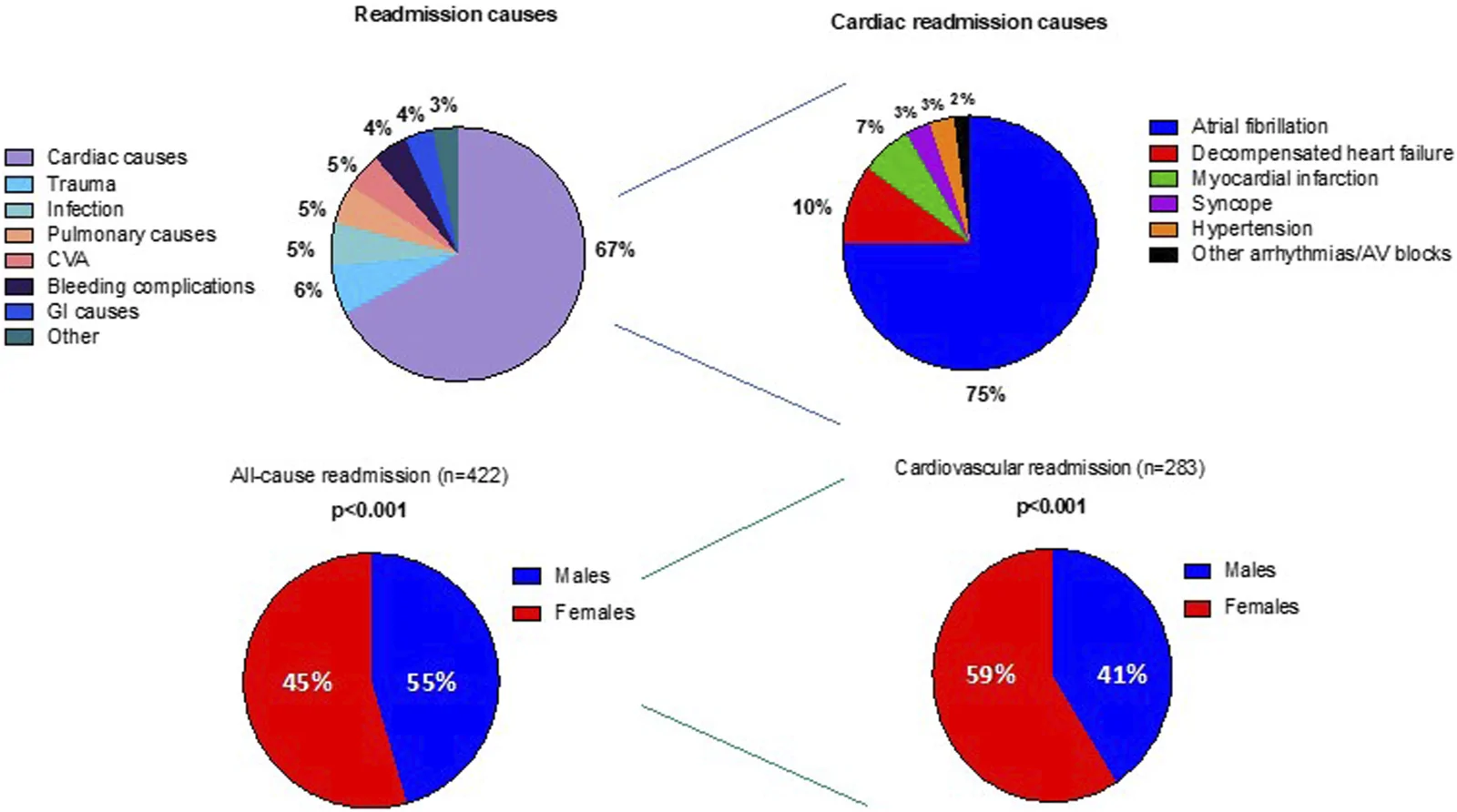
Aetiologies of 1-year readmissions after index admission for atrial fibrillation and stratifications by gender. GI: gastrointestinal. CVA: cerebrovascular event.

### Predictors of 1-year readmission

The Kaplan-Meier analysis demonstrating the relationship between gender and readmissions is shown in Figure 2. We identified that females had an increased risk of readmission compared to males at 1 year (73% vs 56%, *p* < 0.001 for all-cause readmission and 53% vs 34%, *p* < 0.001 for cardiovascular readmission). Similarly, the presence of IHD was linked to an increased risk of all-cause admission at 1 year (63% vs 69%, *p* < 0.001), patients aged ≥60 years at 1 year (42% vs 14%, *p* < 0.001), DM at 1 year (79% vs 60%, *p* < 0.001), and CCF at 1 year (61% vs 77%, *p* < 0.001), as demonstrated by the Kaplan-Meier figures (Figure 2). Univariable Cox regression indicated that patients ≥60 years old (HR: 3.3, 95% CI: 2.7-4.1, *p* < 0.001), females (HR: 1.8, 95% CI: 1.4-2.2, *p* < 0.001), CCF (HR: 2, 95% CI: 1.5-2.7, *p* < 0.001), DM (HR: 1.6, 95% CI: 1.2-2, *p* = 0.03), and IHD (HR: 1.4, 95% CI: 1.1-1.8, *p* = 0.02) were associated with an increased probability of 1-year readmission. Rhythm control with amiodarone was not associated with readmission (HR: 0.9, 95% CI: 0.9-1.3, *p* = 0.44). Multivariate analysis showed that patients ≥60 years (HR: 3.7, 95% CI: 2.9-4.6, *p* < 0.001), DM (HR:1.5 95% CI: 1.2-1.8, *p* = 0.04), and CCF (HR: 3.3, 95% CI: 2.6-4.6, *p* < 0.001) were independently associated with an increased risk of 1-year readmission. Female sex remained independently associated with 1-year readmission after adjustment for these comorbidities (HR 1.7, 95% CI 1.4–2.0, *p* < 0.001) (Table 2).

**Figure 2.**
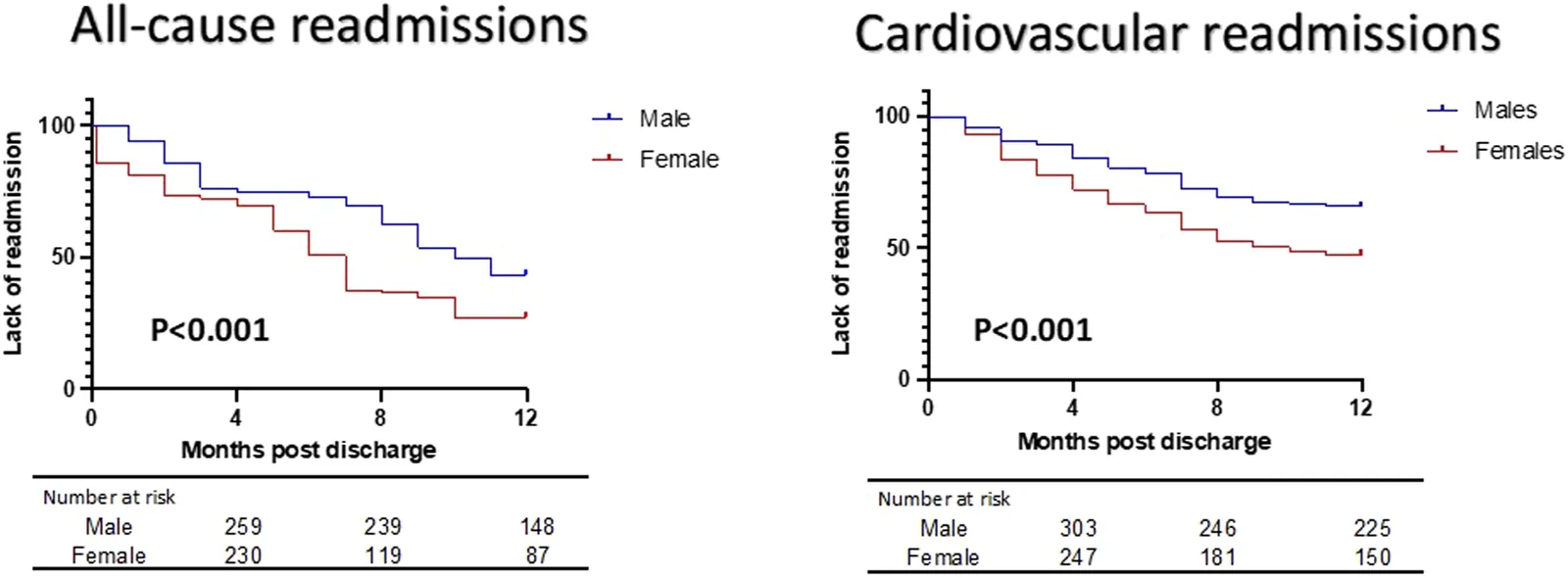
Kaplan-Meier analysis of 1-year all-cause and cardiovascular readmissions in males and females in the study cohort.

**Table 2.** Cox regression showing univariate and multivariable-adjusted predictors of 1-year readmission in the study cohort.

	Univariable analysis		Multivariable- adjusted	
Presenting characteristic	HR (95% CI)	*p*-value	HR (95% CI)	*p*-value
Female vs male	1.8 (1.4–2.2)	<0.001	1.7 (1.4–2)	<0.001
Age ≥60 years (vs < 60 years)	3.3 (2.7–4.1)	<0.001	3.7 (2.9–4.6)	<0.001
Congestive heart failure (yes vs no)	2 (1.5–2.7)	<0.001	3.3 (2.6–4.2)	<0.001
Diabetes mellitus (yes vs no)	1.6 (1.2–2)	0.03	1.5 (1.2–.8)	0.04
Ischemic heart disease (yes vs no)	1.4 (1.1–1.8)	0.02	1.1 (0.8–1.3)	0.13
Cerebrovascular disease (yes vs no)	0.9 (0.7–1.1)	0.18		
Hypertension (yes vs no)	1.1 (0.9–1.)	0.51		
Active thyroid disease (yes vs no)	0.8 (0.4–1.6)	0.29		
Chronic kidney disease (yes vs no)	0.7 (0.5–1.1)	0.33		
Chronic liver disease (yes vs no)	1.3 (0.8–1.6)	0.65		
Active malignancy (yes vs no)	1.1 (0.6–2)	0.48		
Chronic lung disease (yes vs no)	1.3 (0.8–2)	0.22		
Dementia (yes vs no)	0.9 (0.6–1.5)	0.4		
Anaemia (yes vs no)	1.1 (0.7–1.5)	0.37		
Rhythm control (vs rate control)	0.9 (0.7–1.3)	0.44		

HR: hazard ratio. CI: confidence interval.

### Trends and frequencies in readmissions

Figure 3 shows trends in readmissions within 1 year following index admission. Of the patients readmitted, 115 (27%) were readmitted once, 157 (37%) were readmitted twice, and 150 (36%) were readmitted thrice or more. Males had more single readmission compared to females (46% vs 12%, *p* < 0.001), while females had more two and three or more readmissions compared to males (29% vs 44%, *p* < 0.001 and 26% vs 44%, *p* < 0.001, respectively).

**Figure 3.**
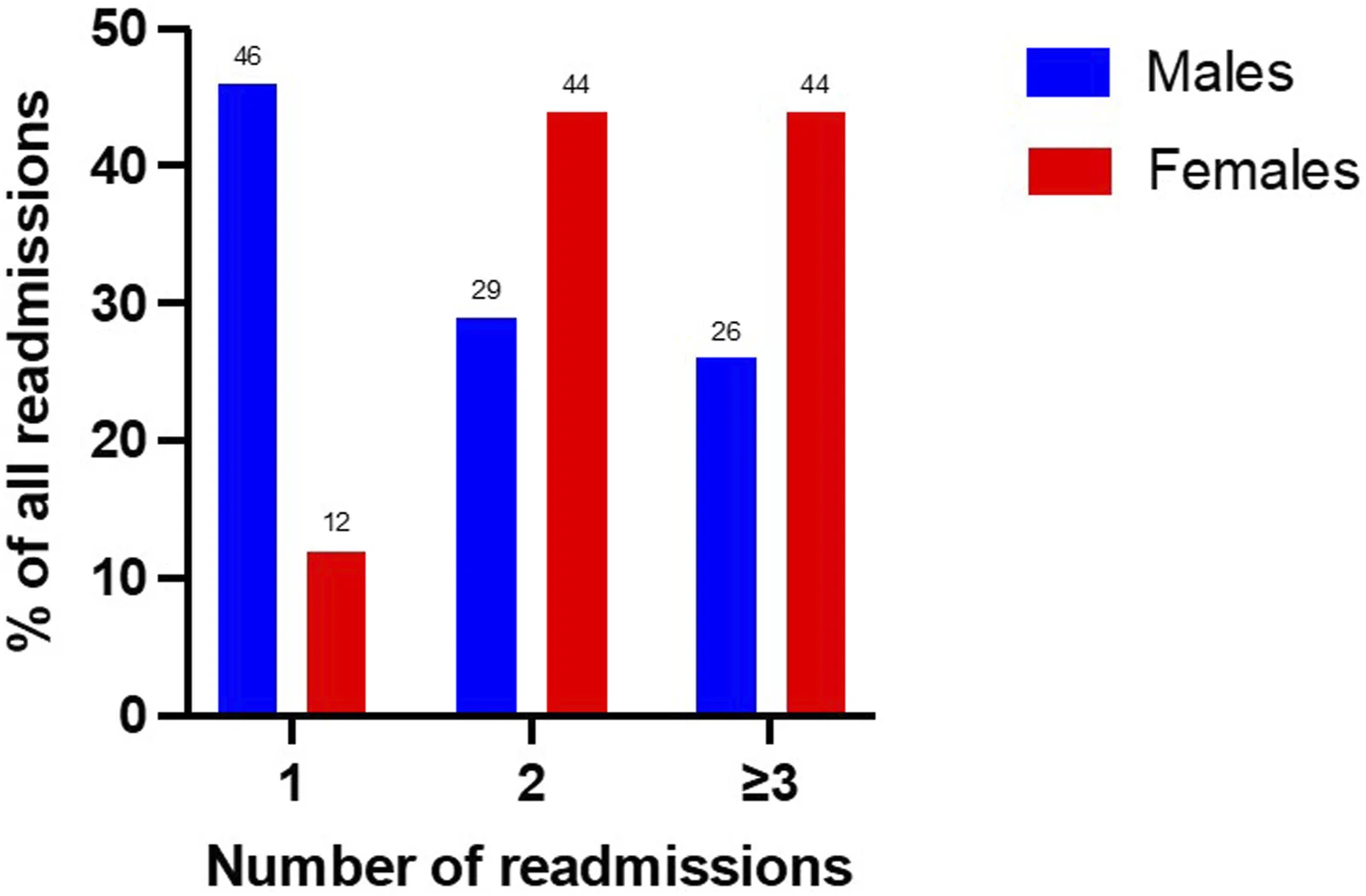
Differences in readmissions in males and females. *p* < 0.001 for all comparisons between males and females.

## Discussion

This study is the first to describe trends and predictors of 1-year readmission following index admission for primary AF in the Middle East and Syria. It highlights several novel findings specific to our Syrian population. First, 64% of Syrian patients were readmitted within 1 year of their index admission, with females being readmitted more than males. Second, DM, CCF, increasing age, and female gender were independently associated with an elevated risk of 1-year readmission. Lastly, females were readmitted more frequently compared to males in this cohort.

As AF has considerable implications on economies worldwide,^17^ recent studies have focused on many aspects of AF, including hospitalisation, quality of life, treatment patterns, and readmission rates.^6,8,13^ Our 1-year readmission rate of 64% significantly exceeds the rates reported in the developed world, at 12.5%.^18^ Furthermore, our 1-year readmission rate exceeds the 2-year readmission rate in Australia, which is 44%.^19^ There was no data from low-to middle-income countries to compare. Our higher readmission rate can be attributed to the ongoing conflict in Syria since 2011, which has primarily impacted health infrastructure and led to a significant turnover of skilled staff, as well as inadequate numbers of nurses and allied health professionals.^20^ As only half the primary health care centres and hospitals are fully functional in Syria,^20^ managing risk factors and following up with patients presenting to hospitals with primary AF after discharge is challenging.

Also, access to medications during conflict has been challenging and should be addressed by international health organisations.^21^ The observed gender differences in readmission risk may reflect biological and sociocultural contributors. Prior studies suggest that women are more likely to experience heart failure with preserved ejection fraction, are more symptomatic, and may metabolise drugs differently.^22,23^ However, in conflict settings such as Syria, systemic gender-based healthcare barriers are also likely contributors. Women may delay seeking care, face logistical or familial constraints, or have reduced access to specialist services. The absence of structured outpatient AF follow-up care may exacerbate these issues. Additionally, factors such as health literacy, socioeconomic status, and family support systems—unavailable in our dataset—likely play roles in post-discharge outcomes. Social determinants of health, including gender norms and caregiving responsibilities, may help explain the higher rate of multiple readmissions among women.

Although similar data existed before the conflict, supporting the Syrian healthcare system, including primary care, would help reduce the 1-year readmission rate in Latakia and nationwide. Our findings provide important insights into gender-specific disparities in AF outcomes in conflict-stricken countries. Females in our cohort had significantly higher rates of both all-cause and cardiovascular readmissions compared to males. This observation persisted even after adjustment for other clinical factors in multivariable analysis, suggesting that female sex is an independent predictor of adverse post-discharge outcomes in this context.

Prior evidence from high-income settings, such as the ORBIT-AF and Euro Heart Survey, has consistently shown that women with AF are more symptomatic, more likely to have heart failure with preserved ejection fraction, and are often under-treated compared to men.^24,25^ In Syria, these disparities may be exacerbated by systemic issues such as limited access to specialist care for women, reduced health-seeking behaviour due to sociocultural factors, and lower prioritisation of women’s cardiovascular health in clinical practice. Moreover, our findings highlight the interaction between biological risk (older age) and structural determinants (access to follow-up care). This suggests that a gendered analysis of health systems performance is urgently needed in fragile settings. The significantly higher proportion of females with recurrent readmissions (two or more) further supports the notion that post-discharge management may be especially inadequate for females.

Addressing these disparities requires better resourcing of health systems and targeted public health policies that promote equitable access to cardiac care, including the provision of structured AF follow-up clinics, guideline-directed therapy, and education of both healthcare providers and patients regarding gender biases in AF care.

Future work should explore interventions that target modifiable risk factors for readmission, particularly among women, and evaluate the impact of structured gender-sensitive post-discharge programmes. Prospective studies are needed to validate these findings and assess whether targeted outpatient management or community-based care models can reduce the burden of recurrent admissions.

These disparities underscore the need for gender-sensitive approaches to AF management. Mitigation strategies may include structured post-discharge follow-up programs, targeted health education, community outreach, and the integration of multidisciplinary care models. Additionally, integrating artificial intelligence (AI) and machine learning may offer new opportunities to enhance risk stratification and personalised care for AF patients in low-resource settings. AI-driven predictive models could help identify patients at greatest risk for readmission—especially among underrepresented groups such as women—using routinely collected clinical data. Such tools could optimise discharge planning, support early interventions, and improve outcomes, even in fragile healthcare systems.

## Limitations

Our study was conducted in a single centre located in Latakia, which has been relatively less affected by direct conflict compared with other regions. Although Tishreen University Hospital functions as a major referral hospital that manages unselected AF patients, including internally displaced individuals, its patient population may differ from those in hospitals located in more heavily damaged or resource-depleted areas. Therefore, the generalisability of our findings to all Syrian regions should be interpreted with caution. This was a single-centre study conducted at Tishreen University Hospital in Latakia. While the hospital is one of the largest referral centres in Syria, serving both residents and displaced patients from conflict-affected regions, our findings may not be fully generalizable to all Syrian hospitals. Regional differences in infrastructure, staffing, and access to cardiovascular care may result in variability in atrial fibrillation management and outcomes across the country. Thus, extrapolation of our results to the wider Syrian population and to other conflict settings should be made with caution. Moreover, our analysis only included data routinely documented in medical records and the number of patients visiting the hospital. Consequently, there may be additional factors influencing mortality that have not been identified. This study did not assess treatments during the initial admission or discharge process, which might have affected the results. Readmissions to centres outside those studied were not included, which may result in an underestimation of the readmission rate. The potential collinearity between older age and female gender may also confound the multivariable regression results, despite statistical adjustment. Granular data on AF subtype, echocardiographic parameters and biomarkers were not consistently available and could not be incorporated into our analyses. These are important predictors of readmission and may represent residual confounders. Our study was limited to admissions at a single tertiary centre. Readmissions to other facilities were not captured and may have led to an underestimation of the true readmission rate. However, as Tishreen University Hospital is the largest referral centre in the region and the main provider of cardiovascular services, most AF-related readmissions were likely recorded in our dataset. Nevertheless, this potential under-capture remains a relevant source of confounding and may have biased our findings. We were unable to capture post-discharge adherence or access to therapy, both of which are likely to influence readmission rates, especially in low-income patients.

## Conclusion

This study highlights a high burden of 1-year unplanned readmissions among patients with AF in a conflict-affected Syrian setting, with females disproportionately affected. Female gender emerged as an independent predictor of readmission, along with older age, DM, and CCF. The increased frequency and severity of readmissions among females point to significant gender disparities in AF outcomes. Addressing these gaps through equitable, gender-sensitive care pathways is essential to improving outcomes in fragile healthcare systems.

## Data Availability

Data relating to this study are available upon reasonable request from the corresponding author.*
